# Supplementary material for: Dysregulation of the Amniotic PPARγ Pathway by Phthalates: Modulation of the Anti-Inflammatory Activity of PPARγ in Human Fetal Membranes
Source: Life (Basel). 2022 Apr 6;12(4):544. doi: 10.3390/life12040544 (PMC9029737; doi:10.3390/life12040544)
Supplement: Supplementary file 1 [file life-12-00544-s001.zip › life-1597598-supplementary.pdf]

Supplementary Material

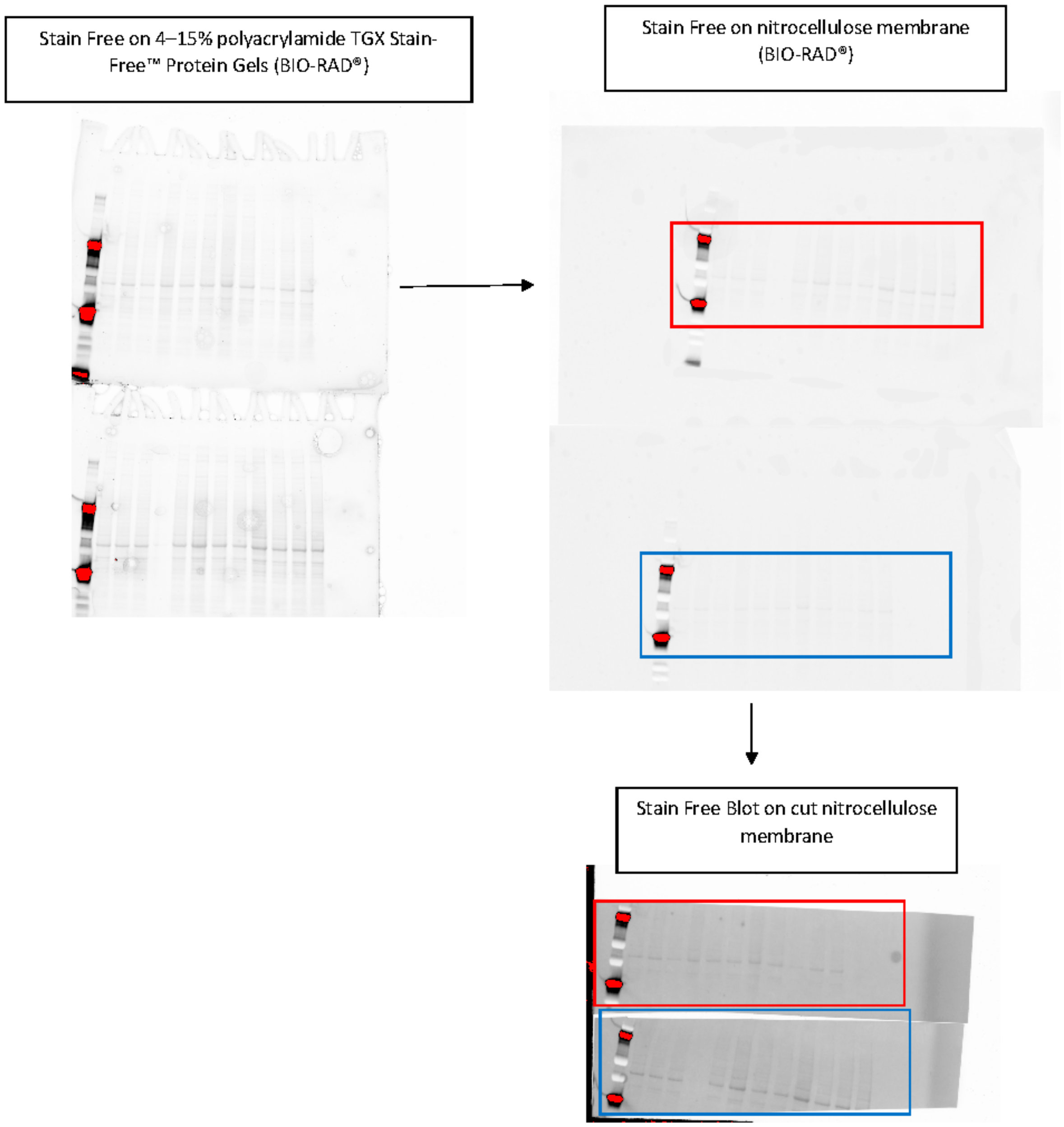

Supplemental figure 1. Western Blot assay steps

After migration of the samples on the 4–15% polyacrylamide gel (BIO-RAD®), the total proteins are visualized by Stain Free gel with ChemiDoc™ Touch Imaging System (BIO-RAD®). After, the transfer of protein on a nitrocellulose membrane, a stain free blot is also realized. For antibody incubation, the membranes are cut in order to save reagents. The membrane peroxidase activity is assayed by enhanced chemiluminescence (Clarity Western ECL Substrate, BIO-RAD®). The relative intensities of the protein bands were analyzed using Image Lab software (BIO-RAD).

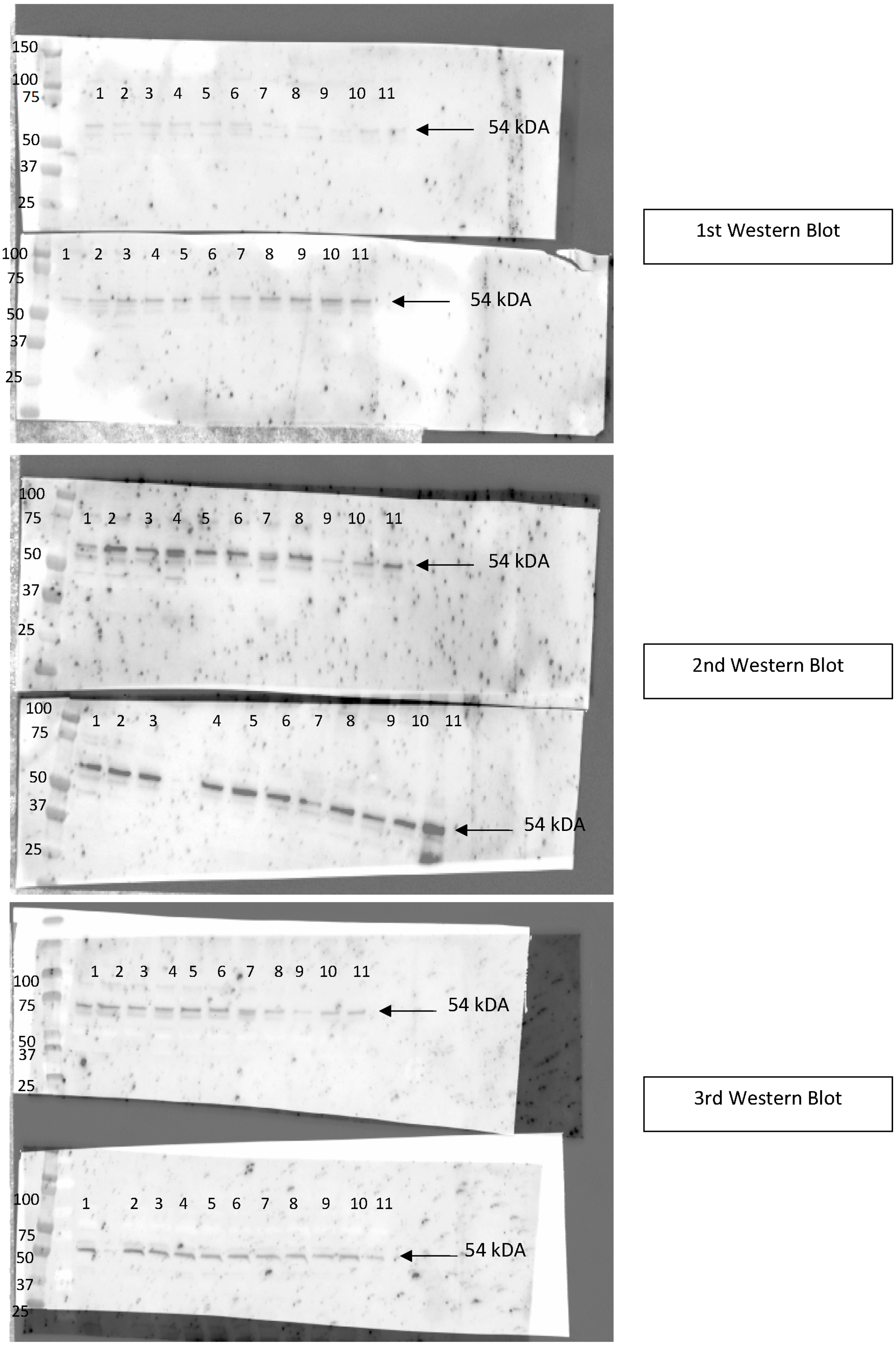

<u>Supplemental figure 2</u>. Western Blot blots and values after integration

For the three blots, upper membrane is for PPARγ protein expression after 24h of treatment; and lower membrane is PPARγ protein expression after 48h of treatment. The relative intensities of the protein bands were analyzed using Image Lab software (BIO-RAD®). The acquisition parameters are kept for all the experiments (high: 6814; low: 5980 and gamma: 1.02). KDa: kilo Daltons.

1: Untreated cells 4: MEHP 10 µM 7: GW9662 1 µM 10: RGT 1 µM + MEHP 10 µM

2: DMSO 5: MEHP 100 µM 8: RGT 1 µM + GW9662 1 µM 11: RGT 1 µM + MEHP 100 µM

3: MEHP 1 µM 6: RGT 1 µM 9 : RGT 1 µM + MEHP 1 µM

3: MEHP 1 µM

	WB 1			WB 2		WB 3	
24	Condition			lane	Norm.Volume	lane	Norm.Volume
	NT	1	98773522	1	19932285	1	13457408
	DMSO	2	104449839	2	23303184	2	13070099
	MEHP 1	3	59835241	3	20975048	3	6006065
	MEHP 10	4	45710269	4	15548484	4	7007212
	MEHP 100	5	67277124	5	12178861	5	12393800
	AGO	6	91885551	6	16351535	6	9797608
	ANTA	7	165395725	7	11832028	7	8581231
	AGO+ANTA	8	135358704	8	14576570	8	5282192
	AGO+MEHP 1	9	159460097	9	3778023	9	4736963
	AGO+MEHP 10	10	116661373	10	7204333	10	13000778
	AGO+MEHP 100	11	118514447	11	9000152	11	10892603

	WB 1			WB 2		WB 3	
48	Condition	lane	Norm.Volume	lane	Norm.Volume	lane	Norm.Volume
	NT	1	18103864	1	18061450	1	18821184
	DMSO	2	19212332	2	18358640	2	20553246
	MEHP 1	3	21109085	3	18879151	3	17210796
	MEHP 10	4	33796485	4	22609948	4	14164194
	MEHP 100	5	40534335	5	18833188	5	9170218
	AGO	6	60677987	6	19543069	6	9691924
	ANTA	7	22499863	7	11491773	7	6812266
	AGO+ANTA	8	42856148	8	11204746	8	8634820
	AGO+MEHP 1	9	41015663	9	13662499	9	11131226
	AGO+MEHP 10	10	39663409	10	18017086	10	9825186
	AGO+MEHP 100	11	21868873	11	22535532	11	4770386
